# Quantitative CT for detecting COVID‑19 pneumonia in suspected cases

**DOI:** 10.1186/s12879-021-06556-z

**Published:** 2021-08-19

**Authors:** Weiping Lu, Jianguo Wei, Tingting Xu, Miao Ding, Xiaoyan Li, Mengxue He, Kai Chen, Xiaodan Yang, Huiyuan She, Bingcang Huang

**Affiliations:** 1 Ningxia Medical University, Yinchuan, 750004 Ningxia China; 2 Department of Radiology, Gongli Hospital, 219 Miaopu Road, Pudong New Area, Shanghai, 200135 China; 3 Department of Infectious Diseases, Gongli Hospital, 219 Miaopu Road, Pudong New Area, Shanghai, 200135 China

**Keywords:** COVID-19, Quantitative CT, Suspected cases, Artificial intelligence, Threshold segmentation

## Abstract

**Background:**

Corona Virus Disease 2019 (COVID-19) is currently a worldwide pandemic and has a huge impact on public health and socio-economic development. The purpose of this study is to explore the diagnostic value of the quantitative computed tomography (CT) method by using different threshold segmentation techniques to distinguish between patients with or without COVID-19 pneumonia.

**Methods:**

A total of 47 patients with suspected COVID-19 were retrospectively analyzed, including nine patients with positive real-time fluorescence reverse transcription polymerase chain reaction (RT-PCR) test (confirmed case group) and 38 patients with negative RT-PCR test (excluded case group). An improved 3D convolutional neural network (VB-Net) was used to automatically extract lung lesions. Eight different threshold segmentation methods were used to define the ground glass opacity (GGO) and consolidation. The receiver operating characteristic (ROC) curves were used to compare the performance of various parameters with different thresholds for diagnosing COVID-19 pneumonia.

**Results:**

The volume of GGO (VOGGO) and GGO percentage in the whole lung (GGOPITWL) were the most effective values for diagnosing COVID-19 at a threshold of − 300 HU, with areas under the curve (AUCs) of 0.769 and 0.769, sensitivity of 66.67 and 66.67%, specificity of 94.74 and 86.84%. Compared with VOGGO or GGOPITWL at a threshold of − 300 Hounsfield units (HU), the consolidation percentage in the whole lung (CPITWL) with thresholds at − 400 HU, − 350 HU, and − 250 HU were statistically different. There were statistical differences in the infection volume and percentage of the whole lung, right lung, and lobes between the two groups. VOGGO, GGOPITWL, and volume of consolidation (VOC) were also statistically different at the threshold of − 300 HU.

**Conclusions:**

Quantitative CT provides an image quantification method for the auxiliary diagnosis of COVID-19 and is expected to assist in confirming patients with COVID-19 pneumonia in suspected cases.

**Supplementary Information:**

The online version contains supplementary material available at 10.1186/s12879-021-06556-z.

## Background

Corona Virus Disease 2019 (COVID-19) is caused by severe acute respiratory syndrome coronavirus (SARS-CoV-2) [1]. It is currently a worldwide pandemic and has a huge impact on public health and socio-economic development. Real-time fluorescence reverse transcription polymerase chain reaction (RT-PCR) detection has widely been used for the diagnosis of COVID-19, but it is time consuming and its false negative rate is high [2, 3]. A lung computed tomography (CT), on the other hand, is easy to perform and has a high sensitivity when diagnosing patients suspected of having COVID-19 [4], especially patients with COVID-19 who initially result negative to RT-PCR [5]. Although some radiology professional organizations and societies, including the Society of Thoracic Radiology (STR), the American College of Radiology (ACR), and the Radiological Society of North America (RSNA), have recommended against performing routine CT for screening and preliminary diagnosis of COVID-19 [6], they play an important role in evaluating severe cases and monitoring disease progression [7–9]. Ground glass opacity (GGO) and consolidation are the most common CT signs of COVID-19 pneumonia [10–12].

In addition, the artificial intelligence (AI) diagnostic system and quantitative evaluation—which is based on CT images—can help doctors quickly come up with a preliminary and differential diagnosis, assess the severity of the disease and predict clinical prognosis [13–19]. The previous study has shown that deep learning-based segmentation systems are highly accurate in automatically delineating lesion regions (a average dice similarity coefficient of 91.6% between automatic and manual segmentations) and lesion percentage metrics (a mean estimation error of 0.3–0.8% on the validation dataset) [20].

However, the definition of GGO and consolidation in conventional CT diagnosis is based on human visual assessment, which also serves as the gold standard for AI automatic delineation training [10–12, 17]. Human visual assessment is arbitrary and can change from person to person and between institutes. Even if some quantitative analysis uses a single CT threshold to segment GGO and consolidation, there is no universal standard [16, 21]. The study of using different CT thresholds to distinguish GGO and consolidation has been completed in pulmonary subsolid nodules [22, 23], but has not yet been applied to COVID-19 pneumonia. The purpose of this study is to define GGO and consolidation based on the segmentation of different CT thresholds, and to apply a quantitative analysis to determine the best parameters for distinguishing between patients with or without COVID-19 pneumonia.

## Methods

### Study design and subjects

Data on patients suspected of having COVID-19 from January 2020 to March 2020 in Shanghai Pudong New Area Gongli Hospital was continuously collected. According to the “Diagnosis and Treatment Protocol for Novel Coronavirus Pneumonia (Trial Version 7)” by National Health Commission of China [2], suspected cases needed to take into account epidemiological history and clinical manifestations (fever and/or respiratory symptoms, normal or decreased white blood cell and lymphocyte count). Inclusion criteria: chest imaging characteristics, including GGO and consolidation; epidemiological history plus any one clinical manifestation, or all two clinical manifestations (if no clear epidemiological history). Image datasets affected by respiratory artifacts in whom image processing and analysis could not be completed were excluded. If the RT-PCR detection resulted positive in coronavirus nucleic acid, it was considered a confirmed case (COVID-19 group). If two consecutive tests gave negative results (sampling time of at least 24 h apart) and the novel coronavirus-specific antibodies IgM and IgG were still negative after 7 days, the case was considered excluded (non-COVID-19 group).

### Acquisition of CT images

Non-contrast enhanced CT examinations were acquired using a multidetector CT scanner with 64 detector rows (LightSpeed VCT, GE Healthcare, USA). The patients were placed in a supine position with their head entering the scanner first. They then inhaled and held their breath. Their lungs—from the entrance of the rib cage to the lower edges of the costal arches on both sides—were scanned all at once. Scanning parameters: tube voltage of 120 kV, tube current of automatic milliamp, collimation width of 40 mm, screw pitch of 0.984, tube rotation time of 0.6 s, layer thickness of 5 mm, layer spacing of 5 mm and matrix of 512 × 512. The high-resolution algorithm reconstructed lung window with 1.25 mm layer thickness, 1200 Hounsfield units (HU) window width and − 600 HU window level. The soft tissue algorithm reconstructed mediastinum window with 1.25 mm layer thickness, 350 HU window width and 40 HU window level.

### Image processing and quantitative analysis

**Fig. 1:**
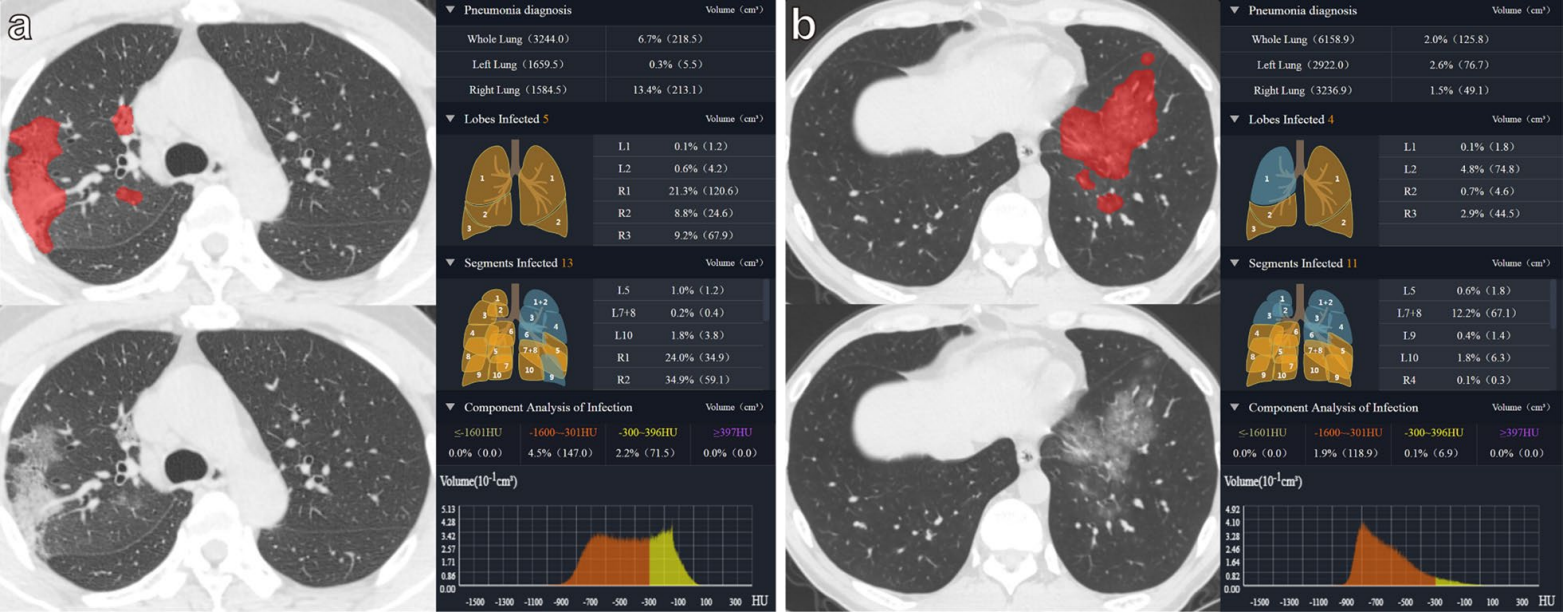
AI automatic lesion extraction and quantitative analysis. **a** 54-years-old man with COVID-19, VOGGO, VOC, GGOPTWL and CPITWL at the threshold of – 300 HU are 147.0 cm^3^, 71.5 cm^3^, 4.5%, 2.2%, respectively; **b** 47-year-old man with non-COVID-19, VOGGO, VOC, GGOPITWL and CPITWL at the threshold of – 300 HU are 118.9 cm^3^, 6.9 cm^3^, 1.9%, and 0.1%, respectively

The automatic identification and quantitative analysis of infection lesions (GGO and consolidation) were performed by an AI imaging diagnosis system (uAI Discover-2019nCoV, Shanghai United Imaging Intelligence Co., Ltd, China). The software used an improved 3D convolutional neural network (VB-Net) to segment the lung CT images, and used the human-in-the-loop (HITL) strategy to iteratively update the model. Specifically, the first segmentation network based on manually contoured CT data was used as an initial model, and then the initial model was applied to the next batch of infection areas, followed by manual correction. In this way of human–machine interaction, the training dataset was iteratively increased, and the final VB-Net model was built [20]. Two expert radiologists in consensus, blinded to clinical information and RT-PCR results, performed artificial contouring and correction. The software finally realised the automatic extraction of the lungs (five lung lobes and 18 lung segments) and the lesion area. It then calculated the parameters, such as the lung lobe segment volume, the infection volume, and the percentage of the infection volume in the lung lobe segment (infection percentage). The CT value distribution of infection lesions was divided into the following eight thresholds: – 500 HU, – 450 HU, – 400 HU, – 350 HU, – 300 HU, – 250 HU, – 200 HU and − 160 HU [22, 23]. The part below the threshold was defined as GGO, the part above the threshold was defined as consolidation. The volume of GGO (VOGGO), GGO percentage in the whole lung (GGOPITWL), volume of consolidation (VOC), consolidation percentage in the whole lung (CPITWL) and GGO percentage in the total lesion (GGOPITTL) were calculated (Fig. 1).

### Statistical methods

Measurement data conforming to the normal distribution was described as Mean ± Standard Deviation (Mean ± SD); measurement data of the skew distribution was described as Median (Md) [Interquartile Range (IQR)]; and counting data was described as composition ratio. Receiver operating characteristic (ROC) curves were performed to evaluate the performance of quantitative parameters at different CT thresholds for diagnosing COVID-19 pneumonia. Using the VOGGO and GGOPITWL at a threshold of – 300 HU as a reference, the DeLong test was used to compare the areas under the curve (AUCs). Baseline information, quantitative CT characteristics and quantitative parameters at a threshold of – 300 HU between COVID-19 and non-COVID-19 groups were analysed using a Mann–Whitney U test for differences. All of the data was statistically analysed using the MedCalc 19.2 (MedCalc Software Ltd, Ostend, Belgium; https://www.medcalc.org) software. A two-tailed *p* value of less than 0.05 was considered statistically significant.

## Results

### Baseline information of the patients

A total of 47 cases—23 males and 24 females—aged from 13 to 81 years, with a median age of 41 years were considered. Among them, 38 cases were in the non-COVID-19 group with a median age of 39 (IQR 21.5) years, a female proportion of 50% and a median time of three (IQR 4.5) days from initial symptom onset to first CT scan. Nine cases were in the COVID-19 group with a median age of 67 (IQR 19) years, a female proportion of 55.56% and a median time of three (IQR 4) days from initial symptom onset to first CT scan. None of the patients had undergone any treatment until the first CT examination. There were statistical differences in age between the two groups, and no statistical differences in gender and time from initial symptom onset to first CT scan. See Table 1 for details.

**Table 1:** Baseline information and quantitative CT characteristics

Characteristics	COVID-19 (n = 9)	Non-COVID-19 (n = 38)	*p*
Age, years (Median [IQR])	67.00 (19.00)	39.00 (21.50)	**< 0.001** *
^#^Female, n (%)	5.00 (55.56)	19.00 (50.00)	1.000
Time from initial symptom onset to first CT scan, days (Median [IQR])	3.00 (4.00)	3.00 (4.50)	0.558
Infection volume (cm^3^), Median (IQR)			
Whole lung	208.70 (347.50)	46.20 (103.20)	**0.017**
Left lung	29.50 (192.70)	15.75 (61.10)	0.417
LUL	8.30 (71.55)	0.85 (13.13)	0.138
LLL	21.60 (113.25)	3.95 (45.90)	0.329
Right lung	146.60 (208.25)	6.85 (49.43)	0.003*
RUL	15.60 (94.35)	0.20 (2.32)	**0.022**
RML	2.20 (16.20)	0.20 (0.50)	**0.019**
RLL	71.40 (149.30)	2.75 (31.03)	0.005*
Infection percentage (%), Median (IQR)			
Whole lung	6.40 (9.85)	1.10 (1.90)	**0.016**
Left lung	1.80 (14.30)	0.75 (3.03)	0.283
LUL	0.80 (6.65)	0.10 (1.08)	0.085
LLL	4.00 (31.15)	0.40 (4.95)	0.167
Right lung	5.10 (13.25)	0.35 (1.58)	0.002*
RUL	2.10 (10.95)	0.00 (0.00)	0.005*
RML	0.50 (5.55)	0.00 (0.00)	0.008*
RLL	9.70 (27.10)	0.25 (2.15)	0.004*

*LUL*  left upper lobe, *LLL *left lower lobe, *RUL* right upper lobe, *RML* right middle lobe, *RLL* right lower lobe

Bold values are statistically significant (*p* < 0.05); *Indicate values of *p* < 0.01

^#^Indicates Fisher’s Exact test; others indicate Mann–Whitney test

### Quantitative CT features

In the COVID-19 group, the infection volume and percentage of the whole lung, right lung, right upper lobe (RUL), right middle lobe (RML), right lower lobe (RLL) were 208.70 (IQR 347.50) cm^3^ and 6.40% (IQR 9.85%), 146.60 (IQR 208.25) cm^3^ and 5.10% (IQR 13.25%), 15.60 (IQR 94.35) cm^3^ and 2.10% (IQR 10.95%), 2.20 (IQR 16.20) cm^3^ and 0.50% (IQR 5.55%), 71.40 (IQR 149.30) cm^3^ and 9.70% (IQR 27.10%), respectively. In the non-COVID-19 group, the above parameter values were 46.20 (IQR 103.20) cm^3^ and 1.10% (IQR 1.90%), 6.85 (IQR 49.43) cm^3^ and 0.35% (IQR 1.58%), 0.20 (IQR 2.32) cm^3^ and 0.00% (IQR 0.00%), 0.20 (IQR 0.50) cm^3^ and 0.00% (IQR 0.00%), 2.75 (IQR 31.03) cm^3^ and 0.25% (IQR 2.15%), respectively. The differences between the two groups were statistically significant (*p* values were 0.017 and 0.016, 0.003 and 0.002, 0.022 and 0.005, 0.019 and 0.008, 0.005 and 0.004, respectively). However, there was no statistical difference in the infection volume and percentage of the left lung, left upper lobe (LUL) and left lower lobe (LLL). See Table 1 for details.

### Quantitative parameter diagnostic efficacy analysis

**Fig. 2:**
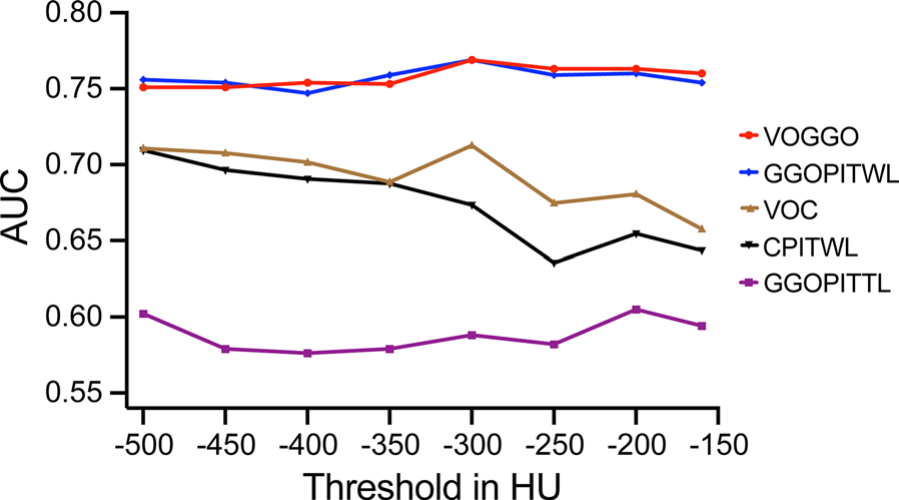
Distribution of AUC values of quantitative parameters at different thresholds

**Fig. 3:**
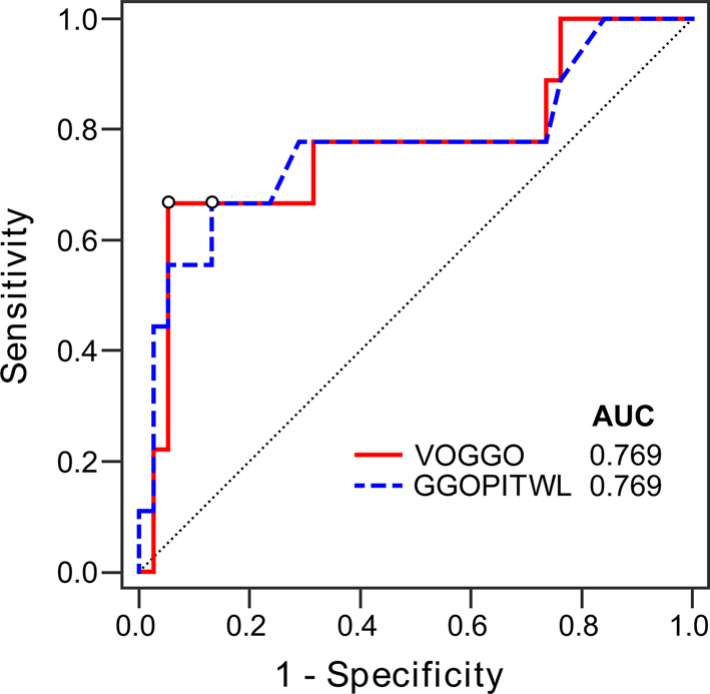
ROC curve analysis for performance of VOGGO and GGOPITWL in diagnosing COVID-19

**Fig. 4:**
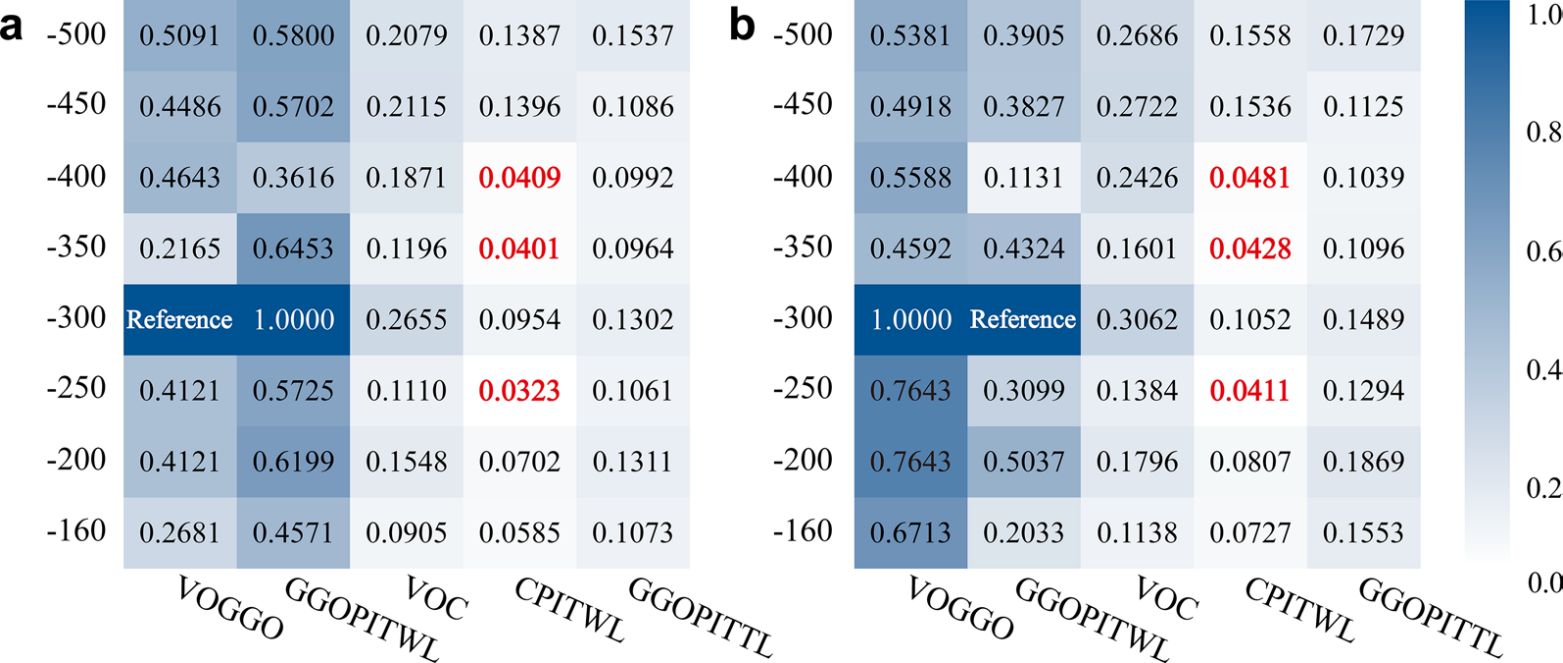
Comparison of AUC values. **a** Comparison between quantitative parameters at different thresholds with VOGGO at a threshold of – 300 HU; **b** Comparison between quantitative parameters at different thresholds with GGOPTWL at a threshold of – 300HU

The ROC curves of quantitative parameters VOGGO, GGOPITWL, VOC, CPITWL and GGOPITTL under eight different thresholds were drawn. An efficacy analysis of the diagnosis of COVID-19 was performed and the respective AUC values were obtained (Table 2). The AUC values of VOGGO and GGOPITWL were higher than the other parameters, with the maximum value being found at the threshold of − 300 HU (Fig. 2). The AUC value of VOGGO was 0.769 (*p* = 0.0124) with sensitivity of 66.67%, specificity of 94.74% and the best cutoff value of > 135.9 cm^3^; the AUC value of GGOPITWL was 0.769 (*p* = 0.0147) with sensitivity of 66.67%, specificity of 86.84% and the best cutoff value of > 2.3% (Table 3; Fig. 3). The CPITWL at thresholds of − 400 HU, − 350 HU, and − 250 HU—compared to the AUC values of VOGGO or GGOPITWL at the threshold of − 300 HU—were statistically different, and there was no difference among others (Fig. 4).

**Table 2:** AUC values of quantitative parameters with different thresholds

Threshold	AUC value of ROC (95% CI)				
(HU)	VOGGO	GGOPITWL	VOC	CPITWL	GGOPITTL
– 500	0.751 (0.604, 0.866)	0.756 (0.609, 0.869)	0.711 (0.560, 0.833)	0.709 (0.558, 0.832)	0.602 (0.449, 0.742)
– 450	0.751 (0.604, 0.866)	0.754 (0.607, 0.868)	0.708 (0.557, 0.831)	0.696 (0.545, 0.822)	0.579 (0.426, 0.721)
– 400	0.754 (0.607, 0.868)	0.747 (0.599, 0.862)	0.702 (0.551, 0.826)	0.690 (0.538, 0.817)	0.576 (0.423, 0.719)
– 350	0.753 (0.605, 0.867)	0.759 (0.612, 0.871)	0.689 (0.537, 0.816)	0.687 (0.535, 0.814)	0.579 (0.426, 0.721)
– 300	0.769 (0.623, 0.879)	0.769 (0.623, 0.879)	0.713 (0.563, 0.836)	0.673 (0.520, 0.802)	0.588 (0.435, 0.729)
– 250	0.763 (0.617, 0.875)	0.759 (0.612, 0.871)	0.675 (0.523, 0.805)	0.635 (0.481, 0.770)	0.582 (0.429, 0.724)
– 200	0.763 (0.617, 0.875)	0.760 (0.613, 0.873)	0.681 (0.529, 0.810)	0.654 (0.501, 0.786)	0.605 (0.452, 0.745)
– 160	0.760 (0.613, 0.873)	0.754 (0.607, 0.868)	0.658 (0.505, 0.790)	0.643 (0.490, 0.778)	0.594 (0.441, 0.734)

*GGO* ground glass opacity, *VOGGO* volume of GGO, *GGOPITWL* GGO percentage in the whole lung, *VOC* volume of consolidation, *CPITWL* consolidation percentage in the whole lung, *GGOPITTL* GGO percentage in the total lesion, *ROC* receiver operating characteristic, *AUC* area under curve, *CI* confidence interval, *HU* Hounsfield units

**Table 3:** Diagnostic performance of COVID-19 (− 300 HU)

Parameters	Cutoff	AUC (95 %CI)	*p*	Sensitivity (%)	Specificity (%)
VOGGO	> 135.9 cm^3^	0.769 (0.623, 0.879)	**0.0124**	66.67	94.74
GGOPITWL	> 2.3 %	0.769 (0.623, 0.879)	**0.0147**	66.67	86.84

Bold values are statistically significant (*p* < 0.05)

### Quantitative parameter difference analysis (threshold of – 300HU)

At a threshold of – 300 HU, the differences of VOGGO (*p* = 0.013), GGOPITWL (*p* = 0.013) and VOC (*p* = 0.048) between the COVID-19 and non-COVID-19 group were statistically significant, while CPITWL (*p* = 0.096) and GGOPITTL (*p* = 0.417) had no statistical difference. See Table 4 for details and Additional file 1: Table S1 for other thresholds.

**Table 4:** Comparison of quantitative parameters between COVID-19 and non-COVID-19 (− 300 HU)

Parameters	COVID-19 (n = 9)	Non-COVID-19 (n = 38)	*p*^#^
VOGGO (cm^3^)	156.80 (320.45)	37.90 (86.68)	**0.013**
GGOPITWL (%)	4.20 (8.30)	0.90 (1.75)	**0.013**
VOC (cm^3^)	23.60 (52.00)	4.85 (15.10)	**0.048**
CPITWL (%)	0.60 (1.70)	0.10 (0.43)	0.096
GGOPITTL (%)	0.91 (0.16)	0.94 (0.17)	0.417

All of data show Median (IQR)

Bold values are statistically significant (*p* < 0.05)

^#^Indicates Mann–Whitney test

## Discussion

A lung CT plays an important role in the screening, diagnosis, evaluation, monitoring and follow-up of COVID-19 patients [4, 5, 7, 24–26]. Our study defined GGO and consolidation by segmenting different CT thresholds, and concluded that the parameters VOGGO and GGOPITWL had the highest diagnostic efficacy when the threshold was at – 300 HU. To the best of our knowledge, this is the first study on quantitative CT analysis which uses different threshold segmentation methods to distinguish between COVID-19 and non-COVID-19 patients in suspected cases. We provide a quantitative method to help doctors diagnose COVID-19 pneumonia.

The study on the application of quantitative CT to COVID-19 pneumonia has been reported in many literatures. Shen et al. [18] believe that quantitative methods can accurately assess the severity and distribution of COVID-19 pneumonia lesions. Lyu et al. [7] also find that the combination of quantitative and qualitative methods can improve the feasibility of the disease severity assessment. Liu et al. [16] find that CT quantitative analysis of lesions can predict the progression to severe diseases at an early stage. In addition, Du et al. [26] applied AI software to quantitative analysis and found that GGO and fibrosis are the main CT features of COVID-19 patients who meet the discharge criteria, and will gradually regress during follow-up. Yu et al. [17] also used quantitative CT analysis to conclude that a large consolidation of the upper lung at admission is associated with a poor prognosis in COVID-19 patients. In contrast, quantitative CT analysis was used to distinguish between COVID-19 and non-COVID-19 patients based on the automatic identification of lesions by AI in our study. The results showed that the infection volume and percentage of the whole lung, right lung and lobes were statistically different between the COVID-19 and non-COVID-19 groups. In addition, at a threshold of – 300 HU, the differences of VOGGO, GGOPITWL and VOC between the two groups were also statistically significant, and the AUC values of VOGGO and GGOPITWL were 0.769 and 0.769, with sensitivity (66.67%, 66.67%) and specificity (94.74%, 86.84%) for diagnosing COVID-19 pneumonia.

There is currently no universal standard for defining CT thresholds for GGO and consolidation. Scholten et al. [22] find that, compared with manual measurements, the semi-automatic measurement of solid components in pulmonary subsolid nodules at a threshold of – 300 HU has very good sensitivity (90%) and specificity (88%). The study of Cohen et al. [23] shows that at a threshold of – 350 HU, the solid components of pulmonary subsolid nodules that automatically segment have no obvious difference from a pathology. The two most effective parameters (VOGGO and GGOPITWL) for diagnosing COVID-19 pneumonia in this study are at a threshold of – 300 HU, which is the same as the segmentation threshold studied by Scholten et al.

This study has the following limitations: first, this is a small sample and single-center study, with age differences between COVID-19 and non-COVID-19 patients, which requires further proper validation in another large scale database; second, GGO and consolidation change with the progression of the disease course [8, 9], but this study fails to carry out a stratified analysis based on the disease development; third, all quantitative analysis is performed on the basis of AI segmentation of lung CT images and automatic extraction of lesions. Therefore, a larger dataset training is required to improve the accuracy of AI.

## Conclusions

Quantitative CT is a promising tool for detecting COVID-19 pneumonia in suspected cases, especially when the CT threshold is at – 300 HU, the quantitative parameters VOGGO and GGOPITWL have a higher specificity in the diagnosis of COVID-19 pneumonia.

## Supplementary information

**Additional file 1: Table S1.** Comparison of quantitative parameters with different thresholds between COVID-19 and non-COVID-19.

## Data Availability

The data and materials are available from the corresponding author on reasonable request.

## Abbreviations

COVID-19: Corona Virus Disease 2019
SARS-CoV-2: Severe acute respiratory syndrome coronavirus
RT-PCR: Reverse transcription polymerase chain reaction
CT: Computed tomography
AI: Artificial intelligence
GGO: Ground glass opacity
VOGGO: Volume of the GGO
GGOPITWL: Percentage of the GGO in the whole lung
VOC: Volume of consolidation
CPITWL: Consolidation percentages of the whole lung
GGOPITTL: GGO percentage in the total lesion
RUL: Right upper lobe
RML: Right middle lobe
RLL: Right lower lobe
LUL: Left upper lobe
LLL: Left lower lobe
HU: Hounsfield units
ROC: Receiver operating characteristic
AUC: Area under the curve
