# Supplementary material for: Quantitative CT for detecting COVID‑19 pneumonia in suspected cases
Source: BMC Infect Dis. 2021 Aug 19;21:836. doi: 10.1186/s12879-021-06556-z (PMC8374412; doi:10.1186/s12879-021-06556-z)
Supplement: Supplementary file 1 — Additional file 1: Table S1. Comparison of quantitative parameters with different thresholds between COVID-19 and non-COVID-19. [file 12879_2021_6556_MOESM1_ESM.docx]

**Table S1** Comparison of quantitative parameters with different thresholds between COVID-19 and non-COVID-19

| Parameters | COVID-19 | Non-COVID-19 | *p*^#^ |
| --- | --- | --- | --- |
|  | (n = 9) | (n = 38) |  |
| Threshold of -500HU |  |  |  |
| VOGGO (cm³) | 105.90 (185.35) | 29.70 (69.10) | **0.020** |
| GGOPITWL (%) | 2.40 (5.40) | 0.70 (1.53) | **0.018** |
| VOC (cm³) | 56.60 (114.10) | 11.30 (29.38) | 0.052 |
| CPITWL (%) | 1.20 (3.70) | 0.20 (0.70) | 0.051 |
| GGOPITTL (%) | 0.76 (0.31) | 0.80 (0.32) | 0.344 |
| Threshold of -450HU |  |  |  |
| VOGGO (cm³) | 112.00 (209.70) | 32.05 (78.80) | **0.019** |
| GGOPITWL (%) | 2.90 (6.10) | 0.80 (1.63) | **0.017** |
| VOC (cm³) | 43.30 (95.60) | 8.80 (25.28) | 0.056 |
| CPITWL (%) | 0.90 (3.05) | 0.20 (0.57) | 0.072 |
| GGOPITTL (%) | 0.83 (0.27) | 0.84 (0.26) | 0.480 |
| Threshold of -400HU |  |  |  |
| VOGGO (cm³) | 116.90 (229.85) | 34.30 (82.73) | **0.019** |
| GGOPITWL (%) | 3.20 (6.70) | 0.85 (1.70) | **0.022** |
| VOC (cm³) | 32.10 (79.10) | 7.35 (22.40) | 0.062 |
| CPITWL (%) | 0.70 (2.55) | 0.10 (0.48) | 0.071 |
| GGOPITTL (%) | 0.86 (0.23) | 0.88 (0.22) | 0.482 |
| Threshold of -350HU |  |  |  |
| VOGGO (cm³) | 122.50 (246.60) | 36.50 (84.98) | **0.019** |
| GGOPITWL (%) | 3.30 (7.20) | 0.90 (1.68) | **0.017** |
| VOC (cm³) | 23.20 (65.05) | 6.20 (19.25) | 0.081 |
| CPITWL (%) | 0.50 (2.05) | 0.10 (0.45) | 0.075 |
| GGOPITTL (%) | 0.89 (0.19) | 0.92 (0.20) | 0.465 |
| Threshold of -300HU |  |  |  |
| VOGGO (cm³) | 156.80 (320.45) | 37.90 (86.68) | **0.013** |
| GGOPITWL (%) | 4.20 (8.30) | 0.90 (1.75) | **0.013** |
| VOC (cm³) | 23.60 (52.00) | 4.85 (15.10) | **0.048** |
| CPITWL (%) | 0.60 (1.70) | 0.10 (0.43) | 0.096 |
| GGOPITTL (%) | 0.91 (0.16) | 0.94 (0.17) | 0.417 |
| Threshold of -250HU |  |  |  |
| VOGGO (cm³) | 152.10 (272.85) | 39.45 (88.05) | **0.015** |
| GGOPITWL (%) | 3.50 (8.10) | 0.95 (1.83) | **0.017** |
| VOC (cm³) | 12.50 (40.05) | 3.50 (12.00) | 0.104 |
| CPITWL (%) | 0.30 (1.25) | 0.10 (0.40) | 0.194 |
| GGOPITTL (%) | 0.94 (0.13) | 0.96 (0.15) | 0.449 |
| Threshold of -200HU |  |  |  |
| VOGGO (cm³) | 164.30 (283.70) | 40.05 (93.00) | **0.015** |
| GGOPITWL (%) | 3.50 (8.40) | 1.05 (1.83) | **0.016** |
| VOC (cm³) | 9.40 (28.30) | 2.20 (9.13) | 0.093 |
| CPITWL (%) | 0.20 (0.90) | 0.00 (0.30) | 0.127 |
| GGOPITTL (%) | 0.96 (0.10) | 0.97 (0.13) | 0.330 |
| Threshold of -160HU |  |  |  |
| VOGGO (cm³) | 166.20 (291.50) | 40.65 (96.23) | **0.016** |
| GGOPITWL (%) | 3.60 (8.75) | 0.95 (1.90) | **0.018** |
| VOC (cm³) | 7.50 (19.35) | 1.90 (7.78) | 0.143 |
| CPITWL (%) | 0.20 (0.60) | 0.00 (0.20) | 0.149 |
| GGOPITTL (%) | 0.97 (0.07) | 0.98 (0.11) | 0.386 |

All Data show Median (IQR); ^#^ indicates Mann-Whitney Test

Bold values are statistically significant (*p* < 0.05)
